# NT157 exerts antineoplastic activity by targeting JNK and AXL signaling in lung cancer cells

**DOI:** 10.1038/s41598-022-21419-6

**Published:** 2022-10-12

**Authors:** Lívia Bassani Lins de Miranda, Keli Lima, Juan Luiz Coelho-Silva, Fabiola Traina, Susumu S. Kobayashi, João Agostinho Machado-Neto

**Affiliations:** 1 Department of Pharmacology, Institute of Biomedical Sciences, University of São Paulo, Av. Prof. Lineu Prestes, 1524, São Paulo, SP 05508-900 Brazil; 2 Laboratory of Medical Investigation in Pathogenesis and Targeted Therapy in Onco-Immuno-Hematology (LIM-31), Department of Hematology, Hospital das Clínicas HCFMUSP, Faculdade de Medicina, University of São Paulo, São Paulo, Brazil; 3 Department of Medical Imaging, Hematology, and Oncology, Ribeirão Preto Medical School, University of São Paulo, Ribeirão Preto, Brazil; 4 Department of Medicine, Beth Israel Deaconess Medical Center and Harvard Medical School, 330 Brookline Avenue, E/CLS-407, Boston, MA 02215 USA; 5 Division of Translational Genomics, Exploratory Oncology Research and Clinical Trial Center, National Cancer Center, Kashiwa, 277-8575 Japan; 6 Harvard Stem Cell Institute, Harvard Medical School, Boston, MA 02215 USA

**Keywords:** Lung cancer, Pharmacology

## Abstract

Combination therapies or multi-targeted drugs have been pointed out as an option to prevent the emergence of resistant clones, which could make long-term treatment more effective and translate into better clinical outcomes for cancer patients. The NT157 compound is a synthetic tyrphostin that leads to long-term inhibition of IGF1R/IRS1-2-, STAT3- and AXL-mediated signaling pathways. Given the importance of these signaling pathways for the development and progression of lung cancer, this disease becomes an interesting model for generating preclinical evidence on the cellular and molecular mechanisms underlying the antineoplastic activity of NT157. In lung cancer cells, exposure to NT157 decreased, in a dose-dependent manner, cell viability, clonogenicity, cell cycle progression and migration, and induced apoptosis (*p* < 0.05). In the molecular scenario, NT157 reduced expression of IRS1 and AXL and phosphorylation of p38 MAPK, AKT, and 4EBP1. Besides, NT157 decreased expression of oncogenes *BCL2, CCND1, MYB*, and *MYC* and increased genes related to cellular stress and apoptosis, *JUN, BBC3, CDKN1A, CDKN1B, FOS*, and *EGR1* (*p* < 0.05), favoring a tumor-suppressive cell signaling network in the context of lung cancer. Of note, JNK was identified as a key kinase for NT157-induced IRS1 and IRS2 phosphorylation, revealing a novel axis involved in the mechanism of action of the drug. NT157 also presented potentiating effects on EGFR inhibitors in lung cancer cells. In conclusion, our preclinical findings highlight NT157 as a putative prototype of a multitarget drug that may contribute to the antineoplastic arsenal against lung cancer.

## Introduction

In the context of personalized therapy, there are few examples of targeted therapy highly selective for a single molecular target that has long-term success, with tyrosine kinase inhibitors for the treatment of chronic myelogenous leukemia being the most successful example^1^. Almost invariably in cases, tumors develop resistance to these therapies using the most diverse strategies, including activation of other signaling pathways that contribute to the maintenance of the malignant phenotype^2^. Thus, the concept of drugs that act on multiple targets to exert their antineoplastic activity has gained prominence in oncology^3,4^.

The compound NT157 belongs to the class of tyrphostin and was initially described as an allosteric inhibitor of insulin-like growth factor 1 receptor (IGF1R). NT157 induces extracellular signal-regulated kinases (ERK)-dependent serine phosphorylation of insulin receptor substrates 1 and 2 (IRS1/2) as a result of shifting IGF1R complexation from IRS1/2 to SHC, thus NT157 leads to long-term inhibition of IGF1R-IRS1/2 signaling^5,6^. Other well-characterized targets of NT157 are the signal transducers and activators of transcription 3 and 5 (STAT3/5), which are inactivated by dephosphorylation through drug-induced protein phosphatase activation^7–9^. Recently, a phosphoproteomic study has shed light on new molecular mechanisms triggered by NT157 that could contribute to its anticancer effects, among them c-Jun N-terminal kinases (JNK) activation and AXL inhibition^10^.

Evidence from genetic interactions and gene expression studies have indicated that IGF1R-IRS1/2 signaling contributes to the development and progression of lung cancer^11–15^. In lung cancer patients, high IGF1R expression was associated with reduced survival outcomes and increased postoperative recurrence^14,16^. Using lung cancer cellular models, IGF1/IGF1R signaling was related to chemo- and radio-resistance^17,18^. About half of lung cancer patients have high expression of STAT3, and constitutive activation of this protein contributes to resistance to conventional therapies^19,20^. In addition, several research groups have associated aberrant AXL activation with therapeutic failure in this disease^21–24^. In the present study, lung cancer was chosen to explore the NT157 multi-target effects, since this disease model contains multiple activated oncogenic pathways relevant to its neoplastic phenotype that are targets of the drug.

## Material and methods

### Cell lines and inhibitors

Lung cancer cell lines NCI-H1299 and NCI-H460 were kindly provided by Dr. Adilson Kleber Ferreira (Institute of Biomedical Sciences of University of São Paulo, São Paulo, Brazil). NCI-H1975 cell line was obtained from the Rio de Janeiro Cell Bank (Rio de Janeiro, Brazil). H1299 and H460 cell lines were cultured in the Roswell Park Memorial Institute medium (RPMI)-1640 and supplemented with 10% fetal bovine serum (FBS), 100 U/ml penicillin, and 100 µg/ml streptomycin, and maintained at 37 °C, 5% CO_2_. H1975 cells were cultured in the RPMI-1640 and supplemented with 2 mM glutamine, 10 mM HEPES, 1 mM sodium pyruvate, 4500 mg glucose, 1500 mg NaHCO_3_, 10% fetal bovine serum (FBS), 100 U/ml penicillin, and 100 µg/ml streptomycin, and maintained at 37 °C, 5% CO_2_. NT157 was obtained from Sun-Shinechem (Wuhan, China). Gefitinib was obtained from Selleck Chemicals (Houston, TX, USA). SP600125 was obtained from Sigma-Aldrich (St. Louis, MO, USA). All drugs were dissolved in dimethyl sulfoxide (DMSO, Sigma-Aldrich), and stored in stock solutions of 10 mM.

### Cell viability assay

Cell viability was determined by a sulforhodamine B (SRB) assay. The lung cancer cells were seeded at the density of 2 × 10^3^ cells per well in a 96-well plate in an RPMI-1640 medium with 10% FBS in the presence of vehicle (Ø) or different concentrations of NT157 (1.6, 3, 6, 12.5, 25, 50 and 100 µM) for 24, 48 and 72 h. For combined treatment, H1299 and H460 cells were exposed to graded concentrations of NT157 (0.4, 0.8, 1.6, 3.2, and 6.4 μM) and gefitinib (3.2, 6.4, 12.5, 25, and 50 μM) alone or in combination with each other for 48 h. Alternatively, H1975 cells were exposed to NT157 (0.8 and 1.6 μM) and gefitinib (12.5 and 25 μM) alone or in combination with each other for 48 h. The cells were then fixed with 10% trichloroacetic acid at 4 °C for at least 1 h. Subsequently, the plates were washed with distilled water three times and a 0.2% solution of SRB diluted in 1% acetic acid was added and the plates were taken to incubate for 30 min at 37 °C. The non-associated dye was removed by washing with 1% acetic acid three times and the plates were dried at room temperature. The plates were then incubated with a 10 mM TRIS pH 10.5 solution under stirring for 30 min at 4 °C. Cell viability was evaluated by measuring the absorbance at 570 nm. The inhibitory concentration (IC)_50_ values were calculated by performing a nonlinear regression analysis in GraphPad Prism 8 (GraphPad Software, Inc., San Diego, CA, USA).

### Colony formation assay

Lung cancer cells were seeded at the density of 1 × 10^3^ cells in 6-well plates, were incubated for 24 h, and then treated with vehicle (Ø) or different concentrations of NT157 (3.2, 6.4 and 12.5 µM). Alternately, H1299 and H460 cells were treated with vehicle (Ø) or SP600125 20 µM for 1 h before the addition of NT157 at 12.5 µM for 6 h and replacement by drug-free medium. Colonies were detected after 7 days of culture by adding 1% crystal violet (Sigma-Aldrich) to a 10% ethanol solution for 15 min at room temperature. Images were acquired using the G:BOX Chemi XRQ (Syngene, Cambridge, UK) and analyzed using ImageJ 1.45 s software (http://imagej.nih.gov/ij; U.S. National Institutes of Health, Bethesda, MD, USA).

### Apoptosis assay

Cell lines were seeded at the density of 2 × 10^6^ cells in 100 mm^2^ plates and after 24 h of incubation were treated with vehicle (Ø) or different concentrations of NT157 (6.4 and 12.5 µM). After 24 h of treatment, the cells were collected and the apoptosis assays were performed by labeling cells with FITC-conjugated annexin V (BD PharMingen, San Diego, CA, USA) and propidium iodide (Thermo Fisher Scientific, San Jose, CA, USA). Samples were incubated for 15 min in the dark at room temperature. For each sample, 10,000 events were acquired on a FACSCalibur (Becton Dickinson, Lincoln Park, NJ, USA) and analyzed with FlowJo software vX.0.7 (Treestar, Inc., San Carlos, CA, USA).

### Mitochondrial membrane potential evaluation by JC-1 stating

H1299 and H460 cells were seeded on 24-well plates and treated with vehicle (Ø) or different concentrations of NT157 (6.4 and 12.5 µM). After 24 h of treatment cells were collected and then washed with PBS and resuspended in RPMI 10% FBS, without phenol red, containing 5 μg/mL JC-1 (BD). After incubation for 30 min at 37 °C and 5% CO_2_ in a light-protected area, viable cells were identified using a forward-scattered light/side-scattered light gate and 10,000 events were acquired by FACSCalibur (Becton Dickinson). Events were analyzed using FlowJo software vX.0.7 (Treestar).

### Cell cycle analysis

A total of 2 × 10^6^ cells were seeded in 100 mm^2^ plates and, after 24 h of incubation, treated with vehicle (Ø) or different concentrations of NT157 (6.4 and 12.5 µM). Cells were fixed in 70% ethanol, for at least 2 h at 4 °C, and stained with 20 μg/mL propidium iodide (PI) containing 10 μg/mL RNase A for 30 min at room temperature. DNA content distribution was acquired using a FACSCalibur (Becton–Dickinson) and analyzed using FlowJo software vX.0.7 (Treestar).

### Immunofluorescence assay

H1299 and H460 cells treated with vehicle (Ø) or different concentrations of NT157 (6.4 and 12.5 µM) for 48 h, were then fixed with ice-cold 100% methanol, permeabilized with 0.5% Triton X-100 in PBS for 30 min at room temperature, and blocked with 1% bovine serum albumin (BSA) in PBS for 1 h at room temperature. Next, the cells were incubated with anti-α-tubulin Alexa Fluor 488 conjugate (cat. no. 53-4502-82; 1:200 in 1% BSA in PBS; Thermo Fisher Scientific) overnight at 4 °C protected from light followed by washing once with PBS. After that, the slides were prepared in ProLong Gold Antifade Mountant with DAPI (Thermo Fisher Scientific) for 1 h at room temperature. Images were captured using a fluorescent microscope (Lionheart FX Automated microscope; Bio-Tek Instruments Inc., Winooski, VT, USA; magnification, × 400).

### Wound healing assay

A total of 4.5 × 10^4^ H1299 cells per well were seeded in a 24-well plate and incubated until they reached 85–95% confluence. The medium was then removed and at the bottom of each well, using a 200 µL pipette, a scratch was performed. PBS was utilized to wash off the debris and fresh medium (RPMI 10% FBS) with vehicle (Ø) or different concentrations of NT157 (6.4 and 12.5 µM) was added. The images were taken periodically (6 and 12 h) on a digital inverted light microscope EVOS AME-3302 (Aeleusden, Netherlands; magnification, × 100).

### Migration assay on spheroid model

A total of 1 × 10^4^ H1299 cells were seeded per well on 96-well plates prepared with 65 μL agarose 1% at the bottom of each well. The cells formed spheroids that incubated for 7 days. Each spheroid was transferred to a 6-well plate and, after they adhered to the plate, they were treated with vehicle (Ø) or different concentrations of NT157 (6.4 and 12.5 µM). The images were taken periodically on a digital inverted light microscope EVOS AME-3302.

### Reverse transcription-quantitative (RT-q) PCR

Total RNA from H1299 and H460 cells treated with vehicle (Ø) or different concentrations of NT157 (6.4 and 12.5 µM) was extracted using TRIzol reagent (Thermo Fisher Scientific) and reverse transcribed using the High-Capacity cDNA Reverse Transcription kit (Thermo Fisher Scientific). Quantitative PCR (qPCR) was performed using a QuantStudio 3 Real-Time PCR System in conjunction with a SybrGreen System (Thermo Fisher Scientific) and specific primers (Supplementary Table 1). *HPRT1* and *ACTB* genes were used as reference genes. The relative quantification value was calculated using the equation 2^−ΔΔCT^ ^25^. A negative ‘No Template Control’ was included for each primer pair.

### Western blotting

H1299 and H460 cells were treated with vehicle (Ø) and NT157 at different concentrations (3.2, 6.4, and 12.5 µM) for 24 h. For combined treatment, cells were treated with vehicle (Ø) or SP600125 20 µM for 1 h before the addition of NT157 at 12.5 µM for 6 h or NT157 (12.5 µM) and/or gefitinib (25 µM) for 24 h, as indicated. Cells were collected and lysed with extraction buffer (10 mM EDTA, 100 mM Tris, 10 nM Na_4_P_2_O_7_, 100 mM NaF, 10 mM Na_3_VO_4_, 2 mM phenylmethane sulfonyl fluoride, 1% Triton X-100). Equal amounts of total protein extract were used, followed by SDS-PAGE, and Western blot analysis with the indicated antibodies (Supplementary Table 2) and the SuperSignal™ West Dura Extended Duration Substrate system (Thermo Fisher Scientific) and G:BOX Chemi XX6 gel document system (Syngene). Cropped gels retain important bands, but whole gel images are available in Supplementary Figs. 1, 2, 3, and 4.

### Statistical analysis

Statistical analyses were performed using GraphPad Prism 8 (GraphPad Software). For comparisons, Student’s t-tests or ANOVA and Bonferroni post-test were used. At least three independent experiments for each method were performed. A *p*-value < 0.05 was considered statistically significant.

## Results

### NT157 reduces cell viability and clonogenicity in lung cancer cells

**Figure 1:**
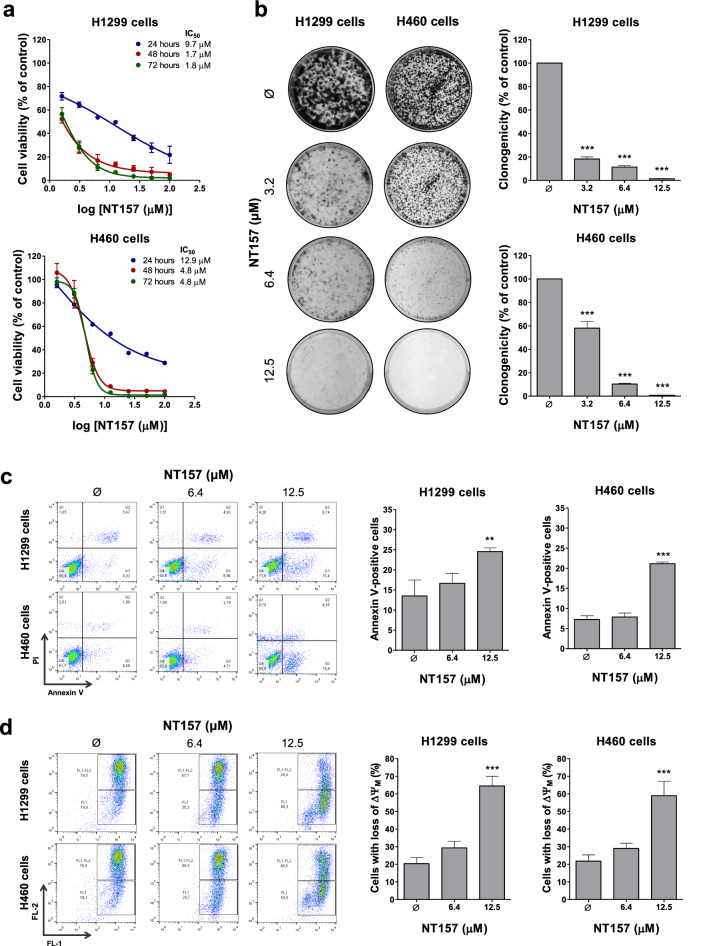
NT157 reduces cell viability, clonogenicity and induces apoptosis in lung cancer cells. (**a**) Dose- and time-response cytotoxicity was evaluated by the sulforhodamine B (SRB) assay. H1299 and H460 cells were treated with vehicle (Ø) or different concentrations of NT157 (1.6, 3.2, 6.4, 12.5, 25, 50, and 100 µM) for 24, 48, and 72 h. Values are expressed as the percentage of viable cells for each condition relative to vehicle-treated cells. Results are shown as mean ± SD of at least 3 independent experiments. (**b**) Colony formation of the cells treated with vehicle or NT157 (1.6, 3.2, 6.4, and 12.5 µM) and for 7 days. The bar graph represents the mean ± SD of the relative number of colonies (% of control). ***p < 0.001; ANOVA and Bonferroni post-test. (**c**) Apoptosis was evaluated through annexin V/PI staining and flow cytometry. H1299 and H460 cells were treated with vehicle or NT157 (6.4 and 12.5 μM). Representative dot plots are shown for each condition; the upper and lower right quadrants (Q2 plus Q3) cumulatively contain the apoptotic population (annexin V + cells). **p < 0.01, ***p < 0.001; ANOVA and Bonferroni post-test. (**d**) Mitochondrial membrane potential analysis was evaluated using the JC-1 staining method and flow cytometry. Lung cancer cells were treated with vehicle or NT157 (6.4 and 12.5 μM). Note that NT157 increased the percentage of cells with disrupted mitochondrial membrane. Representative dot plots are shown for each condition; the gate FL-2 contains cells with intact mitochondria and the gate FL-2/FL-1 contains cells with damaged mitochondria. ***p < 0.001; ANOVA and Bonferroni post-test.

To initiate the study about the effects of NT157 in lung cancer cells, a viability assay was performed. NT157 decreased cell viability in a time- and dose-dependent manner (*p* < 0.05) (Fig. 1a). The IC_50_ ranged from 1.7 to 9.7 μM for H1299 cells and from 4.8 to 12.9 μM for H460 cells. In both cell lines, clonogenicity was strongly reduced in a dose-dependent manner (all *p* < 0.05), and NT157 at 12.5 μM resulted in 1.4% and 0.8% of full capacity to colony formation for H1299 and H460 cells, respectively (Fig. 1b).

### NT157 induces apoptosis and disrupts cell cycle progression in lung cancer cells

**Figure 2:**
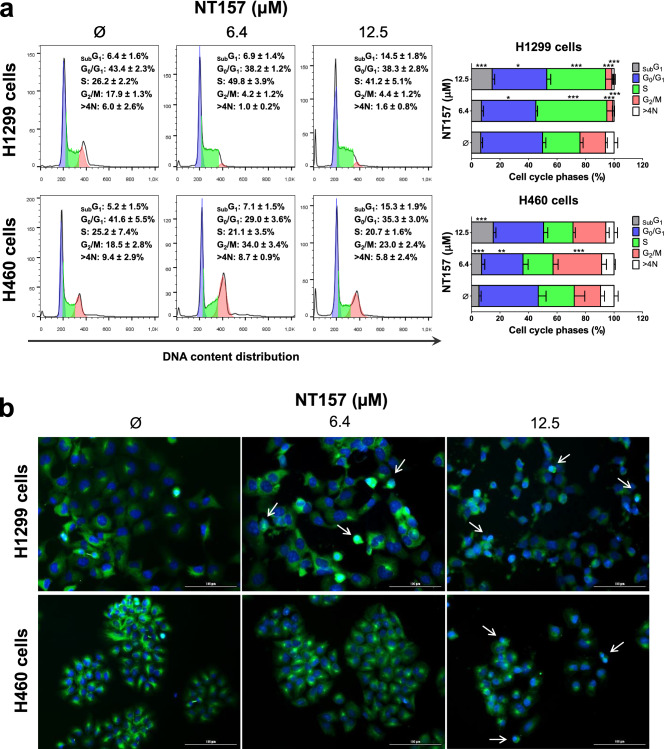
NT157 disrupts the cell cycle in lung cancer cells. (**a**) Cell cycle progression was determined by PI staining and flow cytometry in H1299 and H460 cells treated with vehicle or NT157 (6.4 and 12.5 μM). A representative histogram for each condition is illustrated. Bar graphs represent the mean ± SD of the percent of cells in subG1, G0/G1, S, G2/M, and > 4 N cells. Note that NT157 increased cell population at subG1 for both cell lines. In H1299 and H460 cells, NT157 increased cell population at S and G2/M phases, respectively. *p < 0.05, **p < 0.01 and ***p < 0.001; ANOVA and Bonferroni post-test. (**b**) Immunofluorescence analysis of H1299 and H460 cells treated with vehicle or NT157 (6.4 and 12.5 μM), displaying α-tubulin (green) and DAPI (blue) staining. The white arrows indicate pyknotic nuclei. Scale bar 100 µm.

In H1299 and H460 cells, NT157 induced apoptosis in a dose-dependent manner as observed by phosphatidylserine exposure (Fig. 1c) and loss of mitochondrial membrane potential (Fig. 1d). The increased _sub_G1 cell population and cells with pyknotic nuclei corroborate these findings (Fig. 2a,b). In addition, flow cytometry analysis also indicated that NT157 caused cell cycle disruption in both cell lines, resulting in cycle arrest at the S phase for H1299 cells and at the G_2_/M for H460 (Fig. 2a).

### NT157 reduces cell migration in H1299 cells

**Figure 3:**
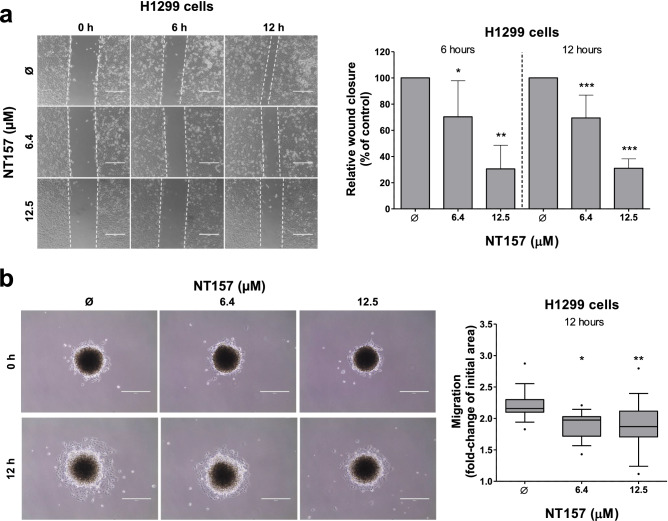
NT157 reduces cell migration on H1299 cells. (**a**) Wound healing assay was performed in H1299 cells treated with vehicle or NT157 (6.4 and 12.5 μM). Images of the cells were obtained at 6 and 12 h. Note that H1299 cells treated with NT157 had a lower wound closure when compared to control. The bar graph represents the mean ± SD of the relative wound closure (% of control). *p < 0.05, **p < 0.01 and ***p < 0.001; ANOVA and Bonferroni post-test. (**b**) Migration assay of a spheroid model derived from H1299 cells treated with vehicle or NT157 (6.4 and 12.5 μM) for 12 h. The final area of the NT157-treated spheroids was smaller than the vehicle-treated spheroids, indicating that the migration was inhibited. Boxplot graph represents the fold-change of the initial migration area. *p < 0.05, **p < 0.01 and ***p < 0.001; ANOVA and Bonferroni post-test.

Using a monolayer migration assay, we observed that NT157 decreased cell migration of H1299 cells in a dose-dependent manner (reduction of approximately 30 and 70% at 6.4 and 12.5 μM, respectively; *p* < 0.05) (Fig. 3a). Additionally, cell migration was also performed using a tridimensional model in H1299 cells, which revealed that NT157-treated spheroids migrate less when compared to vehicle-treated spheroids (*p* < 0.05, Fig. 3b).

### NT157 downregulates IGF1R/IRS and AXL signaling and JNK contributes to NT157-induced IRS phosphorylation in lung cancer cells

**Figure 4:**
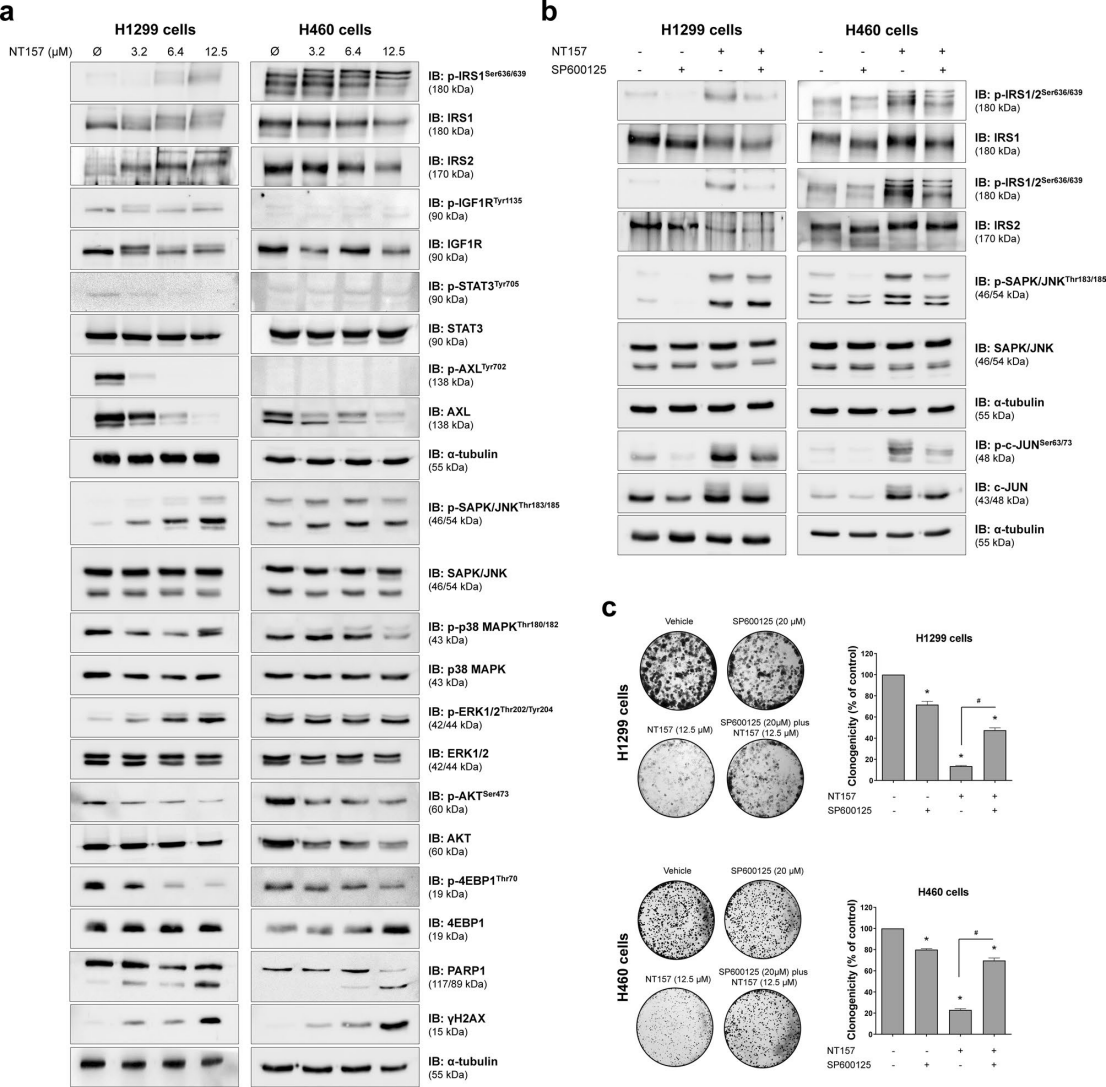
NT157 modulates IGF1R/IRS, JNK, and AXL signaling in lung cancer cells. (**a**) Western blot analysis for p-IRS1^Ser636/639^, IRS1, IRS2, p-IGF1R^Tyr1136^, IGF1R, p-STAT3^Tyr705^, STAT3, p-AXL^Tyr702^, AXL, p-SAPK/JNK^Thr183/185^, SAPK/JNK, p-p38 MAPK^Thr180/182^, p38 MAPK, p-ERK1/2^Thr202/Tyr204^, ERK1/2, p-AKT^Ser473^, AKT, p-4EBP1^Thr70^, 4EBP1, PARP1, and γH2AX in total cell extracts from H1299 and H460 cells treated with vehicle or NT157 (3.2, 6.4, and 12.5 μM) for 24 h. (**b**) Western blot analysis of indicated protein involved in IRS1/2-JNK axis for H1299 and H460 cells treated with vehicle, SP600125 (20 μM) and/or NT157 (12.5 μM) for 6 h. Note that NT157 induces IRS1 and IRS2 phosphorylation, which is prevented by SP600125. Expression of p-c-JUN^Ser63/73^ was used as a control for SP600125 efficacy. Membranes were reprobed with the antibody for the detection of the respective total protein or α-tubulin, and developed with the SuperSignal™ West Dura Extended Duration Substrate system using a G:BOX Chemi XX6 gel doc imaging system. (**c**) H1299 and H460 cells were treated with vehicle (Ø) or SP600125 20 µM for 1 h before the addition of NT157 at 12.5 µM for 6 h and replacement by drug-free medium. Colony formation was evaluated after 7 days of incubation. The bar graph represents the mean ± SD of the relative number of colonies (% of control). *p < 0.05 for SP600125-treated and/or NT157-treated cells vs. vehicle cells, ^#^*p* < 0.05 for SP600125-treated or NT157-treated cells vs. combination treatment at the corresponding doses, ANOVA and Bonferroni post-test.

In the molecular scenario, NT157 reduced expression of IRS1 and AXL and phosphorylation of p38 MAPK, AKT, and 4EBP1 (Fig. 4a). Considering that AXL is highly expressed in lung cancer and impacts therapeutic response^21–24^, we further analyzed the effects of NT157 on this target. AXL expression is higher in H1299 cells compared to H460 cells and repression of AXL expression by NT157 occurs at the mRNA level (Supplementary Fig. 5). NT157 also increased apoptosis and DNA damage markers, PARP1 cleavage and H2AX phosphorylation, respectively. In H1299, but not H460 cells, ERK phosphorylation was increased by NT157. Of note, JNK phosphorylation was increased by NT157 in both cell lines (Fig. 4a).

Previous studies indicated that IRS1 and IRS2 phosphorylation triggered by NT157 is induced by ERK, but this molecular phenomenon was not observed in some cellular models^9^, suggesting that other MAPK may be involved in the process. JNK, similarly to ERK, are also able to phosphorylate IRS1 and IRS2, therefore indicating that it may be related to IRS phosphorylation. To assess this hypothesis the compound SP600125, a selective JNK inhibitor, was used alone or in combination with NT157. As expected, NT157 induced IRS1 and IRS2 phosphorylation, activated JNK, and increased the expression of c-JUN. On the other hand, pharmacological JNK inhibition prevented NT157-induced IRS1 and IRS2 phosphorylation in both lung cancer cells studied (Fig. 4b). Reduced c-JUN phosphorylation confirmed the efficiency of SP600125. At the functional level, short exposure to JNK inhibitor attenuated the antineoplastic effects of NT157 in lung cancer cells (Fig. 4c). Taken together, our data indicated that JNK has a key role in the NT157-induced IRS1 and IRS2 phosphorylation, revealing a novel axis involved in the mechanism of action of the drug.

### NT157 favors a tumor-suppressive gene expression network in lung cancer cells

**Figure 5:**
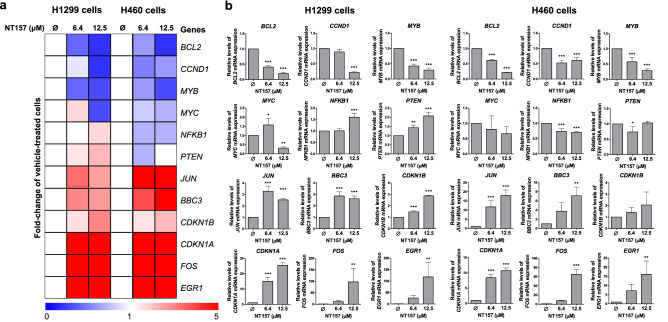
NT157 decreases the expression of oncogenes and induces expression of cellular stress- and apoptosis-related genes in lung cancer cells. (**a**) Heatmap of the gene expression in H1299 and H460 cells treated with vehicle or NT157 (6.4 and 12.5 μM). The data are represented as the fold-change of vehicle-treated cells, and downregulated and upregulated genes are shown by blue and red colors, respectively. (**b**) The bar graphs represent the mean ± SD of at least three independent experiments. *p < 0.05, **p < 0.01 and ***p < 0.001, ANOVA and Bonferroni post-test.

Using qPCR assay for known genes modulated NT157 in other cancer models^9,26,27^, we identified that NT157 decreased expression of oncogenes *BCL2, CCND1, MYB*, and *MYC* and increased genes related to cellular stress and apoptosis, *JUN, BBC3, CDKN1A, CDKN1B, FOS*, and *EGR1* (all *p* < 0.05, Fig. 5). On the other hand, H1299 cells had an increase of *NFKB1* and *PTEN* expression, which was the opposite effect that happened to H460 cells (*p* < 0.05). These results indicated that NT157 favors a tumor-suppressive gene expression network in H1299 and H460 cells.

### NT157 potentiates the reduction of cell viability induced by EGFR inhibitor in lung cancer cells

**Figure 6:**
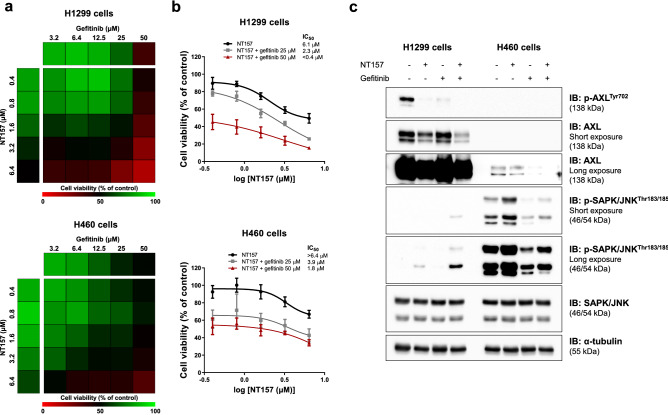
NT157 and gefitinib have potentiating antineoplastic effects on lung cancer cells. (**a**) The heatmap indicates the cell viability of H1299 and H460 cells treated with graded concentrations of NT157 (0.4, 0.8, 1.6, 3.2, and 6.4 μM) and gefitinib (3.2, 6.4, 12.5, 25, and 50 μM) alone or in combination with each other for 48 h, as indicated. Increased cell viability is indicated by green color, while reduced cell viability is indicated by red color. (**b**) Dose–response cytotoxicity for NT157 in combination with gefitinib (25 or 50 μM) for H1299 and H460 cells. Values are expressed as the percentage of viable cells for each condition relative to untreated controls. Results are shown as the mean of at least three independent experiments. (**c**) Western blot analysis of p-AXL^Tyr702^, AXL, p-SAPK/JNK^Thr183/185^, SAPK/JNK in H1299 and H460 cells treated with vehicle, NT157 (12.5 μM) and/or gefitinib (25 μM) for 24 h. Membranes were reprobed with the antibody for the detection of the respective total protein or α-tubulin, and developed with the SuperSignal™ West Dura Extended Duration Substrate system using a G:BOX Chemi XX6 gel doc imaging system.

Finally, since overactivation of IGF1R and AXL have been associated with resistance to EGFR inhibitors^23,28^, we investigated the effects of the combination between NT157 and gefitinib. Of note, NT157 in combination with gefitinib presented potentiating effects in the reduction of cell viability of H1299 and H460 cells (Fig. 6a,b). At the molecular level, gefitinib potentiates NT157-induced JNK phosphorylation in H1299 cells, and reduction of AXL in H1299 and H460 cells (Fig. 6c).

## Discussion

Herein, we have investigated the cellular and molecular underlying the antineoplastic effects of NT157 in lung cancer cells. NT157 is a synthetic compound initially described as an inhibitor of IGF1R-IRS1/2^5,6^, but currently, new targets of this compound have been identified, including STAT3/5 and AXL^7,9,10^. In lung cancer cells, exposure to NT157 decreased cell viability, clonogenicity, cell cycle progression and migration, and induced apoptosis, which corroborates previous findings in diseases models of hematological neoplasms^9,26^ and others solid tumors^5,8,29–31^. In fact, lung cancer has become an interesting model to elucidate the mechanism of action of NT157 since, in this model, signaling mediated by IGF1R-IRS1/2, STAT3, and AXL has already been reported as relevant to the development, progression, and therapeutic response of this type of cancer^14,32^. Of note, overexpression of AXL has been reported and associated with resistance to therapy in lung cancer^21–24^. Thus, the identification of compounds that act simultaneously in multiple oncogenic pathways may be interesting to avoid the emergence of resistant clones.

In the molecular scenario of the present study, NT157 inhibited IRS1/2 and its downstream targets (AKT and 4EBP1), reduced p38 MAPK phosphorylation and AXL expression, increased JNK phosphorylation and markers of cell death and DNA damage (cleavage of PARP1 and yH2AX)^33,34^. In H1299 cells (more sensitive to NT157), but not in H460, NT157 reduced phosphorylation of IGF1R and STAT3. Among these findings, we highlight the significant reduction in AXL. In a previous study, NT157-induced AXL reduction was identified through proteomic studies, but evidence of *AXL* reduction at the gene level was not observed^10^. In our study, *AXL* mRNA levels were reduced in H1299 cells, which have higher expression of this gene, suggesting a transcriptional regulation. Of note, a direct association between IGF1R and AXL pathways is not reported, but tumor cells expressing low AXL levels exhibit high levels of IGF1R phosphorylation^35^. In addition, AXL-mediated signaling has multiple downstream targets in common with the IGF1R-mediated signaling pathway, including MAPK, JAK2/STAT, and PI3K/AKT/mTOR^36^. Thus, the simultaneous inhibition of both receptors could generate a more prominent inhibition of these downstream targets and prevent the activation of reactivation loops of the pathway.

Activation of ERK1/2 was the first signaling axis described as relevant for NT157-induced phosphorylation, inhibition, and degradation of IRS proteins^6^. However, later studies in hematological malignancies found that NT157 inhibits IRS1/2 even in the absence of ERK phosphorylation^9^, indicating that another underlying mechanism could be involved. In the present study, ERK activation was observed only in H1299 cells, but IRS1/2 phosphorylation and degradation were observed in both cell lines studied, suggesting that other MAPK could be involved. JNK is a MAPK that phosphorylates IRS1/2 in the inhibitory domain, similarly to ERK^37^. Using the selective JNK inhibitor, SP600125, we demonstrated that this MAPK is essential for the inhibition of IRS proteins, independent of the modulation of ERK, elucidating a new signaling axis involved in the mechanism of action of NT157.

NT157 also decreased p38 MAPK activation, which despite being classically associated with cellular stress in other cell types, is frequently expressed and related to cell proliferation in lung cancer^38,39^. Similarly, AKT and 4EBP1, downstream targets of the PI3K/AKT/mTOR pathway, were inhibited by NT157, further highlighting the antineoplastic potential of this compound in lung cancer, since this signaling pathway is mutated or aberrantly activated in this disease^40,41^. Although the initial characterization of the cellular and molecular effects of NT157 was performed in KRAS-mutated lung cancer cells, key findings of the present study were also observed in EGFR-mutated lung cells, including a dose-dependent reduction in cell viability and clonogenicity, reduction in AXL-mediated signaling, JNK activation and potentiating effects in combination therapy with gefitinib (Supplementary Fig. 6).

Our gene expression analysis indicated that NT157 efficiently reduced the expression of several oncogenes and enhanced the expression of different genes related to cell cycle arrest and apoptosis in both cell lines. NT157 reduced BCL2 expression, a potent anti-apoptotic protein^42^, as well as the cell cycle regulator *CCND1*, commonly overexpressed in cancer^43^, and the proto-oncogenes *MYB* and *MYC*, which have a relevant role in tumor growth, proliferation, and motility in lung cancer^44,45^. In both lung cancer cells, NT157 increased *JNK* and *FOS* expression, genes related to AP-1 complex and cellular stress^46^, as well as *BBC3*, a proapoptotic gene upregulated by p53^47^. Additionally, NT157 exposure enhanced the expression of *CDKN1A* and *CDKN1B*, which encode respectively to p21 and p27, proteins that act as cell cycle regulators, preventing cycle progression^48,49^. In both cell lines, *EGR1* expression was enhanced upon NT157 exposure, which is a tumor suppressor that inhibits cell migration and induces apoptosis in non-small cell lung cancer^50^. On the other hand, NT157 induced opposite effects on the expression of PTEN and NFKB1 in H1299 and H460 cells. The function of NFKB1 in the cancer context is still not completely understood and has been linked from inflammation and tumor development to the induction of apoptosis^51^. The function of PTEN is well established, being the tumor suppressor commonly inhibited in tumors and one of the main negative regulators of the PI3K/AKT/mTOR pathway^52^. Taken together, these findings indicate that NT157 acts on multiple targets and favors a tumor suppressor cell signaling network in the context of lung cancer.

In summary, our results indicate that NT157 has multiple antineoplastic effects in lung cancer cell models. JKN activation induced by NT157 represents a novel mechanism involved in IRS phosphorylation and inhibition induced by the drug. Our preclinical findings highlight NT157 as a putative prototype of a multitarget drug that may contribute to the antineoplastic arsenal against lung cancer.

## Supplementary Information

Supplementary Information 1.

Supplementary Information 2.

## Data Availability

The datasets used and/or analyzed during the current study are available from the corresponding author upon reasonable request.
