# Supplementary material for: NT157 exerts antineoplastic activity by targeting JNK and AXL signaling in lung cancer cells
Source: Sci Rep. 2022 Oct 12;12:17092. doi: 10.1038/s41598-022-21419-6 (PMC9556623; doi:10.1038/s41598-022-21419-6)
Supplement: Supplementary file 1 — Supplementary Information 1. [file 41598_2022_21419_MOESM1_ESM.pdf]

NT157 exerts antineoplastic activity by targeting JNK and AXL signaling in lung cancer cells

Lívia Bassani Lins de Miranda[1], Keli Lima[1,2], Juan Luiz Coelho-Silva[3], Fabiola Traina[3], Susumu S. Kobayashi[4,5,6], João Agostinho Machado-Neto[1,*]

[1] Department of Pharmacology, Institute of Biomedical Sciences, University of São Paulo, São Paulo, Brazil
[2] Laboratory of Medical Investigation in Pathogenesis and Targeted Therapy in Onco-Immuno-Hematology (LIM-31), Department of Hematology, Hospital das Clínicas HCFMUSP, Faculdade de Medicina, University of São Paulo, São Paulo, Brazil
[3] Department of Medical Imaging, Hematology, and Oncology, Ribeirão Preto Medical School, University of São Paulo, Ribeirão Preto, Brazil
[4] Department of Medicine, Beth Israel Deaconess Medical Center and Harvard Medical School, 330 Brookline Avenue, E/CLS-407, Boston, MA 02215, USA
[5] Division of Translational Genomics, Exploratory Oncology Research and Clinical Trial Center, National Cancer Center, Kashiwa 277-8575, Japan
[6] Harvard Stem Cell Institute, Harvard Medical School, Boston, MA 02215, USA

List of supplementary material

Supplementary Table 1. Primer sequences and concentrations.
Supplementary Table 2. List of antibodies for western blot.
Supplementary Figure 1. Whole gel images from Western blot analysis.
Supplementary Figure 2. Whole gel images from Western blot analysis.
Supplementary Figure 3. Whole gel images from Western blot analysis.
Supplementary Figure 4. Whole gel images from Western blot analysis.
Supplementary Figure 5. Downregulation of AXL induced by NT157 occurs at the mRNA level in lung cancer cells.
Supplementary Figure 6. NT157 displays antineoplastic activity in H1975, an EGFR-mutated cell line.

Supplementary Table 1. Primer sequences and concentrations.

Gene	Sequence	Concentration
AXL	FW: GTGGGCAACCCAGGGAATATC<br>RV: GTACTGTCCCGTGTCGGAAAG	300 nM
BBC3	FW: GACCTCAACGCACAGTACGAG<br>RV: AGGAGTCCCATGATGAGATTG	300 nM
BCL2	FW: ATGTGTGTGGAGAGCGTCAA<br>RV: ACAGTTCCACAAAGGCATCC	300 nM
CCND1	FW: CTCGGTGTCCTACTTCAAATG<br>RV: AGCGGTCCAGGTAGTTCAT	300 nM
CDKN1A	FW: TGTCACTGTCTTGTACCCTTGT<br>RV: GCCGGCGTTTGGAGTGGTAG	300 nM
CDKN1B	FW: ACTCTGAGGACACGCATTTGGT<br>RV: TCTGTTCTGTTGGCTCTTTTGTT	300 nM
ERG1	FW: CTTCAACCCTCAGGCGGACA<br>RV: GGAAAAGCGGCCAGTATAGGT	300 nM
FOS	FW: AGAATCCGAAGGGAAAGGAA<br>RV: CTTCTCCTTCAGCAGGTTGG	300 nM
JUN	FW: CAGGTGGCACAGCTTAAACA<br>RV: GTTTGCAACTGCTGCGTTAG	300 nM
MYB	FW: CTCCGCCTACAGCTCAACTCC<br>RV:TCCTTTATTCGCTTTTCCTTCTCA	300 nM
MYC	FW: GCCCCTGGTGCTCCATGA<br>RV: TTCCACAGAAACAACATCGATT	300 nM
NFKB1	FW: GGCAGCACTACTTCTTGACC<br>RV: CAGCAAACATGGCAGGCTAT	300 nM
PTEN	FW: TCCCAGTCAGAGGCGCTATG<br>RV: CACAAACTGAGGATTGCAAG	300 nM
HPRT1	FW: GAACGTCTTGCTCGAGATGTGA<br>RV: TCCAGCAGGTCAGCAAAGAAT	150 nM
ACTB	FW: AGGCCAACCGCGAGAAG<br>RV: ACAGCCTGGATAGCAACGTACA	150 nM

Supplementary Table 2. List of antibodies for western blot.

Manufacturer	Target	Specie	Catalog
Cell Signaling Technology (MA, USA)	p-IRS1$^{Ser636/639}$	Rabbit	#2388
	IRS1	Rabbit	#3407
	IRS2	Rabbit	#3089
	p-IGF1R^{Tyr1135}	Rabbit	#3918
	IGF1R	Rabbit	#3027
	p-STAT3^{Tyr705}	Rabbit	#9131
	STAT3	Rabbit	#4904
	p-AXL^{Tyr702}	Rabbit	#5724
	AXL	Rabbit	#4566
	p-SAPK/JNK$^{Thr183/185}$	Rabbit	#9251
	SAPK/JNK	Rabbit	#9252
	p-p38 MAPK$^{Thr180/182}$	Rabbit	#9211
	p38 MAPK	Rabbit	#9212
	p-ERK1/2$^{Thr202/Tyr204}$	Rabbit	#9101
	ERK1/2	Rabbit	#9102
	p-AKT^{Ser473}	Rabbit	#4060
	AKT	Rabbit	#4685
	p-4EBP1^{Thr70}	Rabbit	#9455
	4EBP1	Rabbit	#9452
	p-c-JUN$^{Ser63/73}$	Rabbit	#3270
	c-JUN	Rabbit	#9165
	PARP1	Rabbit	#9542
	α-tubulin	Rabbit	#2144
Santa Cruz Biotechnology (Santa Cruz, USA)	γH2AX	Mouse	sc-517348

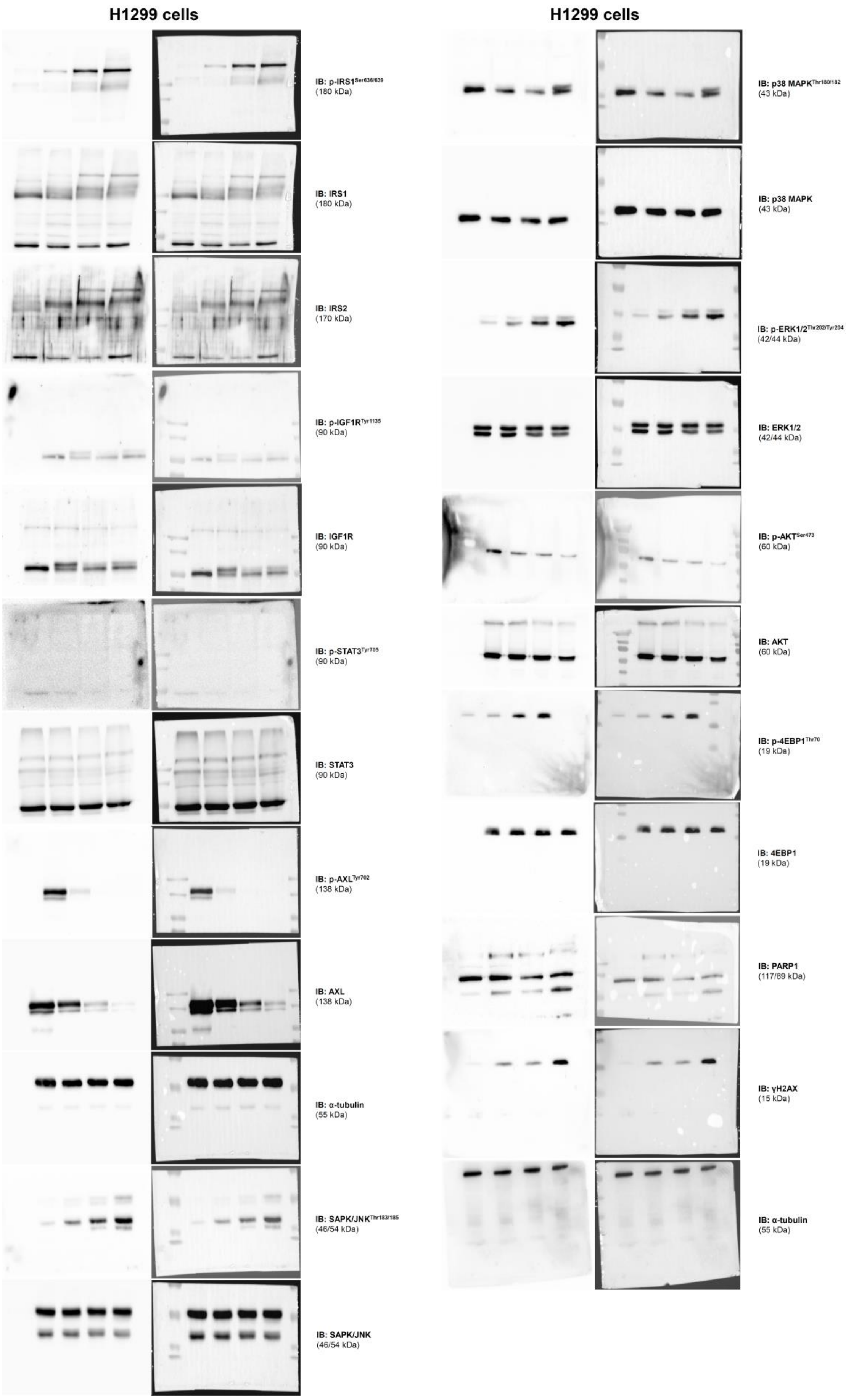
H1299 cells
IB: p-IRS1$^{Ser636/639}$
(180 kDa)
IB: IRS1
(180 kDa)
IB: IRS2
(170 kDa)
IB: p-IGF1R^{Tyr1135}
(90 kDa)
IB: IGF1R
(90 kDa)
IB: p-STAT3^{Tyr705}
(90 kDa)
IB: STAT3
(90 kDa)
IB: p-AXL^{Tyr702}
(138 kDa)
IB: AXL
(138 kDa)
IB: α-tubulin
(55 kDa)
IB: SAPK/JNK$^{Thr183/185}$
(46/54 kDa)
IB: SAPK/JNK
(46/54 kDa)
H1299 cells
IB: p38 MAPK$^{Thr180/182}$
(43 kDa)
IB: p38 MAPK
(43 kDa)
IB: p-ERK1/2$^{Thr202/Tyr204}$
(42/44 kDa)
IB: ERK1/2
(42/44 kDa)
IB: p-AKT^{Ser473}
(60 kDa)
IB: AKT
(60 kDa)
IB: p-4EBP1^{Thr70}
(19 kDa)
IB: 4EBP1
(19 kDa)
IB: PARP1
(117/89 kDa)
IB: γH2AX
(15 kDa)
IB: α-tubulin
(55 kDa)

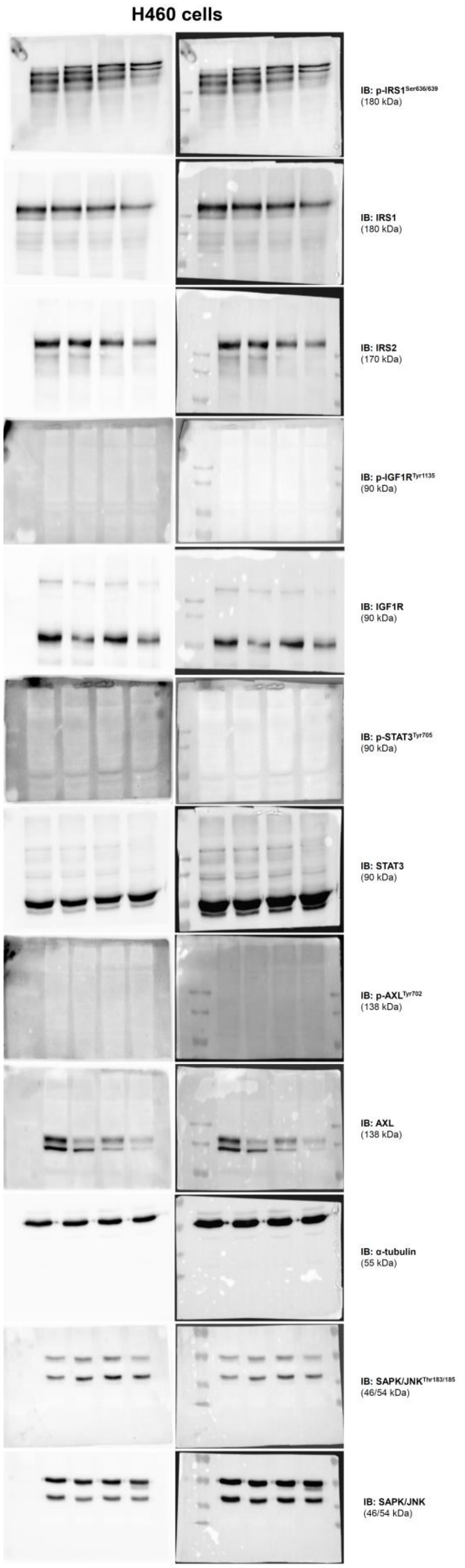
H460 cells
IB: p-IRS1$^{Ser636/639}$
(180 kDa)
IB: IRS1
(180 kDa)
IB: IRS2
(170 kDa)
IB: p-IGF1R^{Tyr1135}
(90 kDa)
IB: IGF1R
(90 kDa)
IB: p-STAT3^{Tyr705}
(90 kDa)
IB: STAT3
(90 kDa)
IB: p-AXL^{Tyr702}
(138 kDa)
IB: AXL
(138 kDa)
IB: α-tubulin
(55 kDa)
IB: SAPK/JNK$^{Thr183/185}$
(46/54 kDa)
IB: SAPK/JNK
(46/54 kDa)

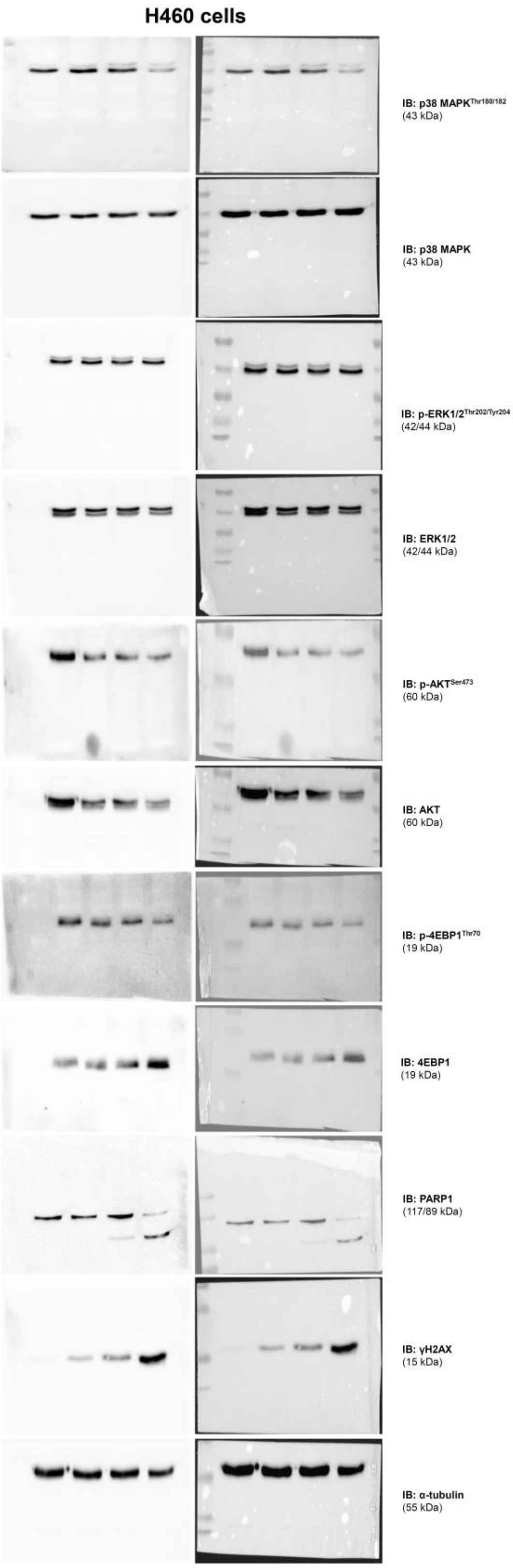
H460 cells
IB: p38 MAPK$^{Thr180/182}$
(43 kDa)
IB: p38 MAPK
(43 kDa)
IB: p-ERK1/2$^{Thr202/Tyr204}$
(42/44 kDa)
IB: ERK1/2
(42/44 kDa)
IB: p-AKT^{Ser473}
(60 kDa)
IB: AKT
(60 kDa)
IB: p-4EBP1^{Thr70}
(19 kDa)
IB: 4EBP1
(19 kDa)
IB: PARP1
(117/89 kDa)
IB: γH2AX
(15 kDa)
IB: α-tubulin
(55 kDa)

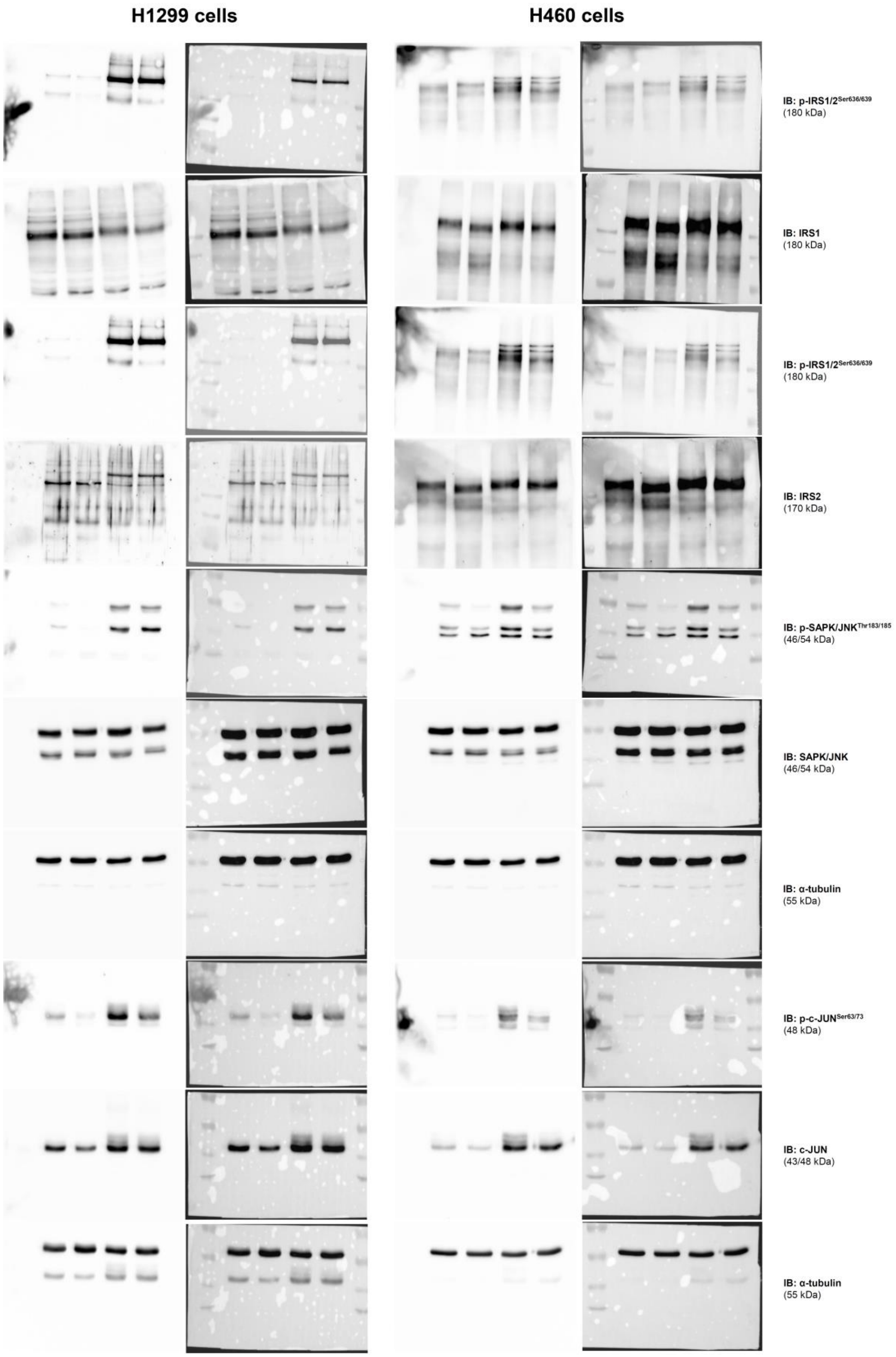

H1299 cells
H460 cells
IB: p-IRS1/2Ser636/639
(180 kDa)
IB: IRS1
(180 kDa)
IB: p-IRS1/2Ser636/639
(180 kDa)
IB: IRS2
(170 kDa)
IB: p-SAPK/JNKThr183/185
(46/54 kDa)
IB: SAPK/JNK
(46/54 kDa)
IB: α-tubulin
(55 kDa)
IB: p-c-JUNSer63/73
(48 kDa)
IB: c-JUN
(43/48 kDa)
IB: α-tubulin
(55 kDa)

Supplementary Figure 1. Whole gel images from Western blot analysis. Western blot analysis in cell extracts from H1299 and H460 cells under experimental conditions (see Figure 4). Membranes were reprobed with the antibody for the detection of the respective total protein or α-tubulin, and developed with the SuperSignal™ West Dura Extended Duration Substrate system using a G:BOX Chemi XX6 gel doc imaging system.

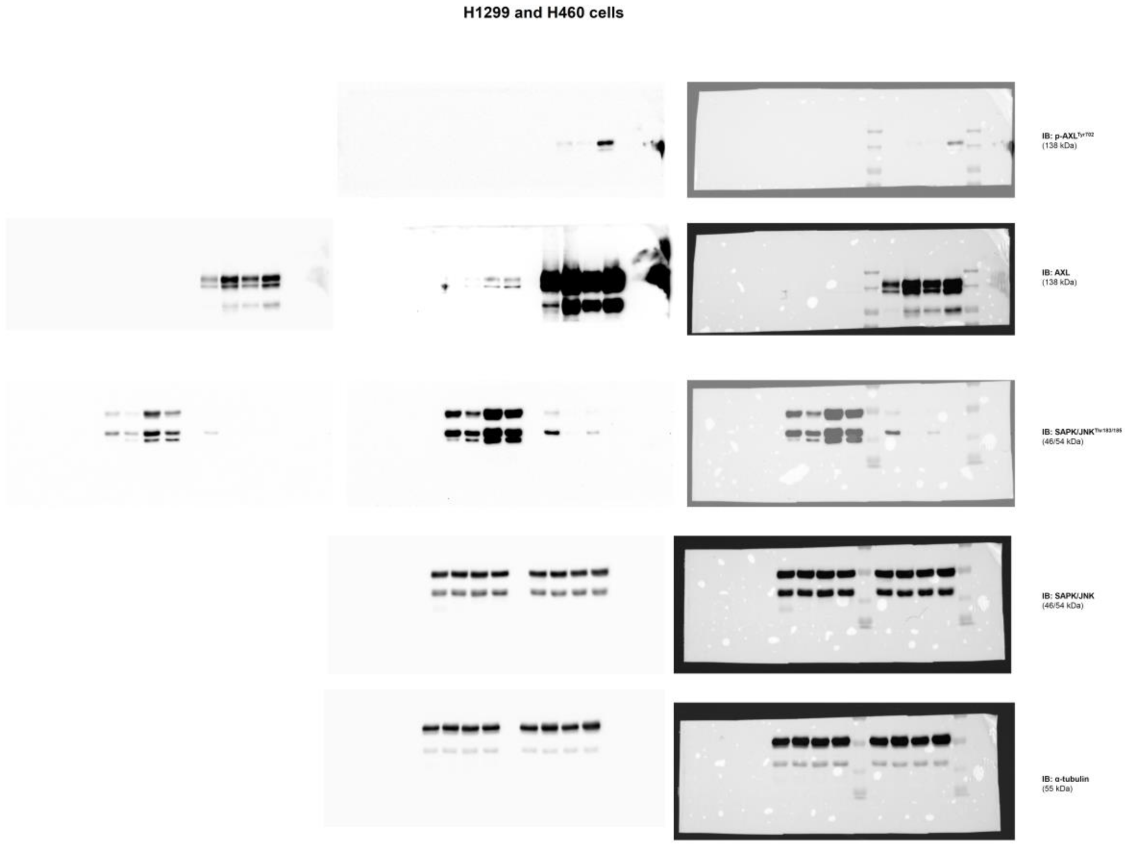

Supplementary Figure 2. Whole gel images from Western blot analysis. Western blot analysis in cell extracts from H1299 and H460 cells under experimental conditions (see Figure 6). Membranes were reprobed with the antibody for the detection of the respective total protein or α-tubulin, and developed with the SuperSignal™ West Dura Extended Duration Substrate system using a G:BOX Chemi XX6 gel doc imaging system.

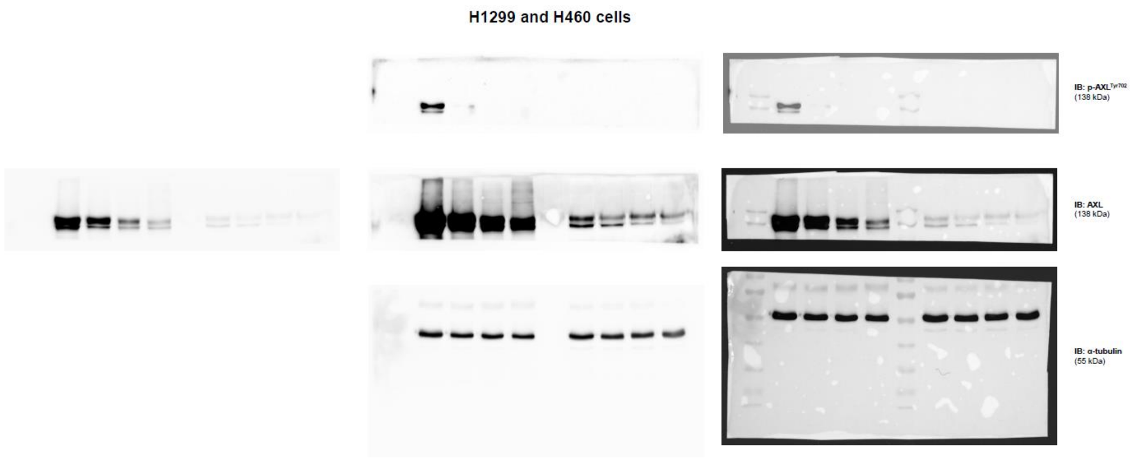

Supplementary Figure 3. Whole gel images from Western blot analysis. Western blot analysis in cell extracts from H1299 and H460 cells under experimental conditions (see Supplementary Figure 5). Membranes were reprobed with the antibody for the detection of the respective total protein or α-tubulin, and developed with the SuperSignal™ West Dura Extended Duration Substrate system using a G:BOX Chemi XX6 gel doc imaging system.

H1975 cells

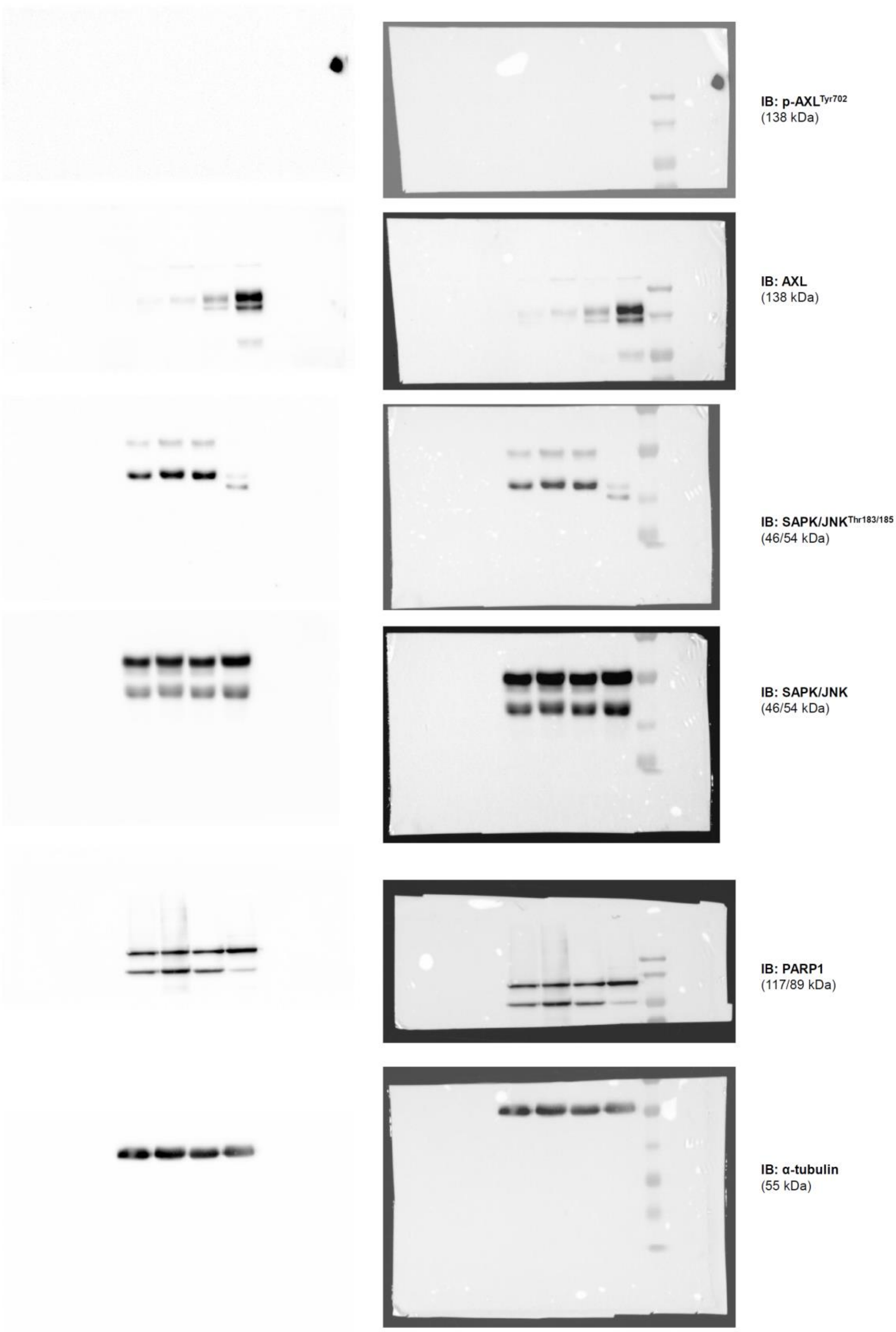

Supplementary Figure 4. Whole gel images from Western blot analysis. Western blot analysis in cell extracts from H1299 and H460 cells under

experimental conditions (see Supplementary Figure 6). Membranes were reprobed with the antibody for the detection of the respective total protein or α-tubulin, and developed with the SuperSignal™ West Dura Extended Duration Substrate system using a G:BOX Chemi XX6 gel doc imaging system.

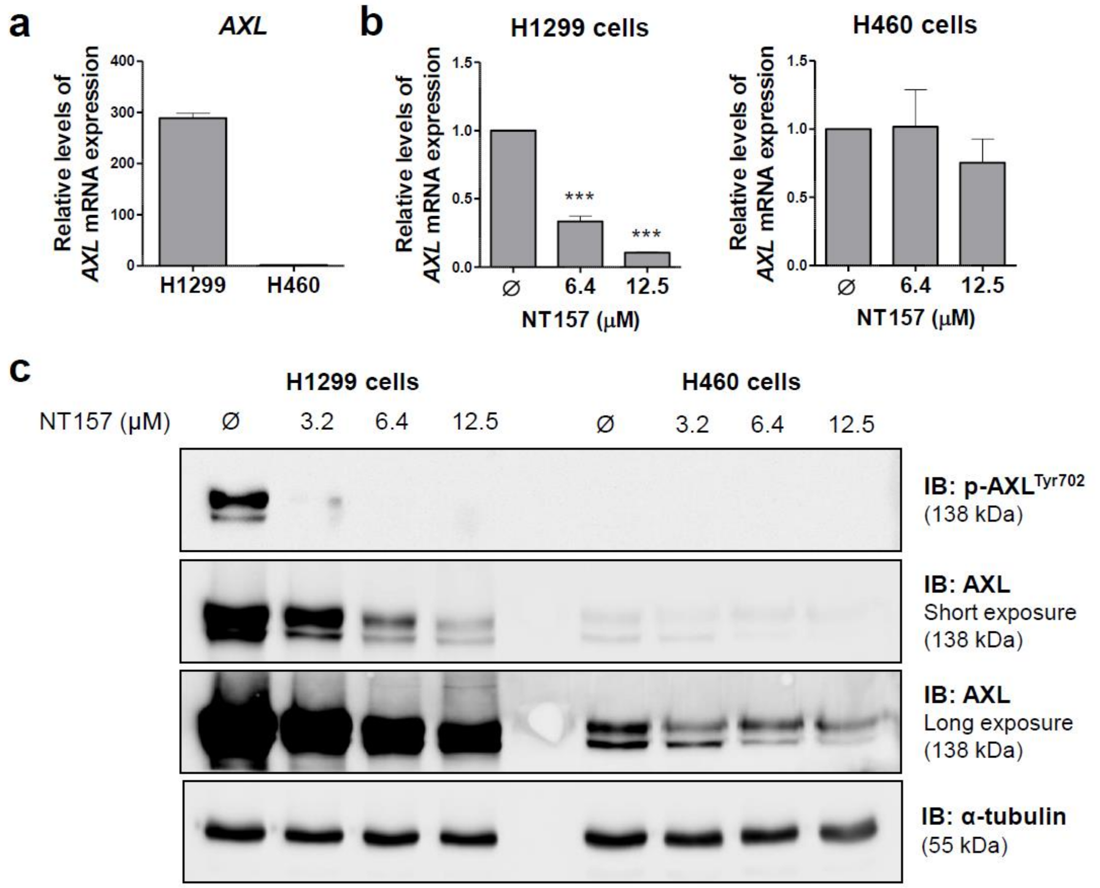

Supplementary Figure 5. Downregulation of AXL induced by NT157 occurs at the mRNA level in lung cancer cells. *AXL* mRNA levels were evaluated by qPCR in H1299 *versus* H460 cells **(a)** or cells treated with vehicle or NT157 (3.2, 6.4, and 12.5 μM) **(b)**. The bar graph represents the mean ± SD of at least three independent experiments. ****p* < 0.001, ANOVA and Bonferroni post-test. **(c)** Western blot analysis for p-AXL^{Tyr702} and AXL in total cell extracts from H1299 and H460 cells treated with vehicle or NT157 (3.2, 6.4, and 12.5 μM) for 24 hours. Membranes were reprobed with an antibody to detect α-tubulin, and images were then developed with a SuperSignal™ West Dura Extended Duration Substrate system using a G:BOX Chemi XX6 gel doc imaging system.

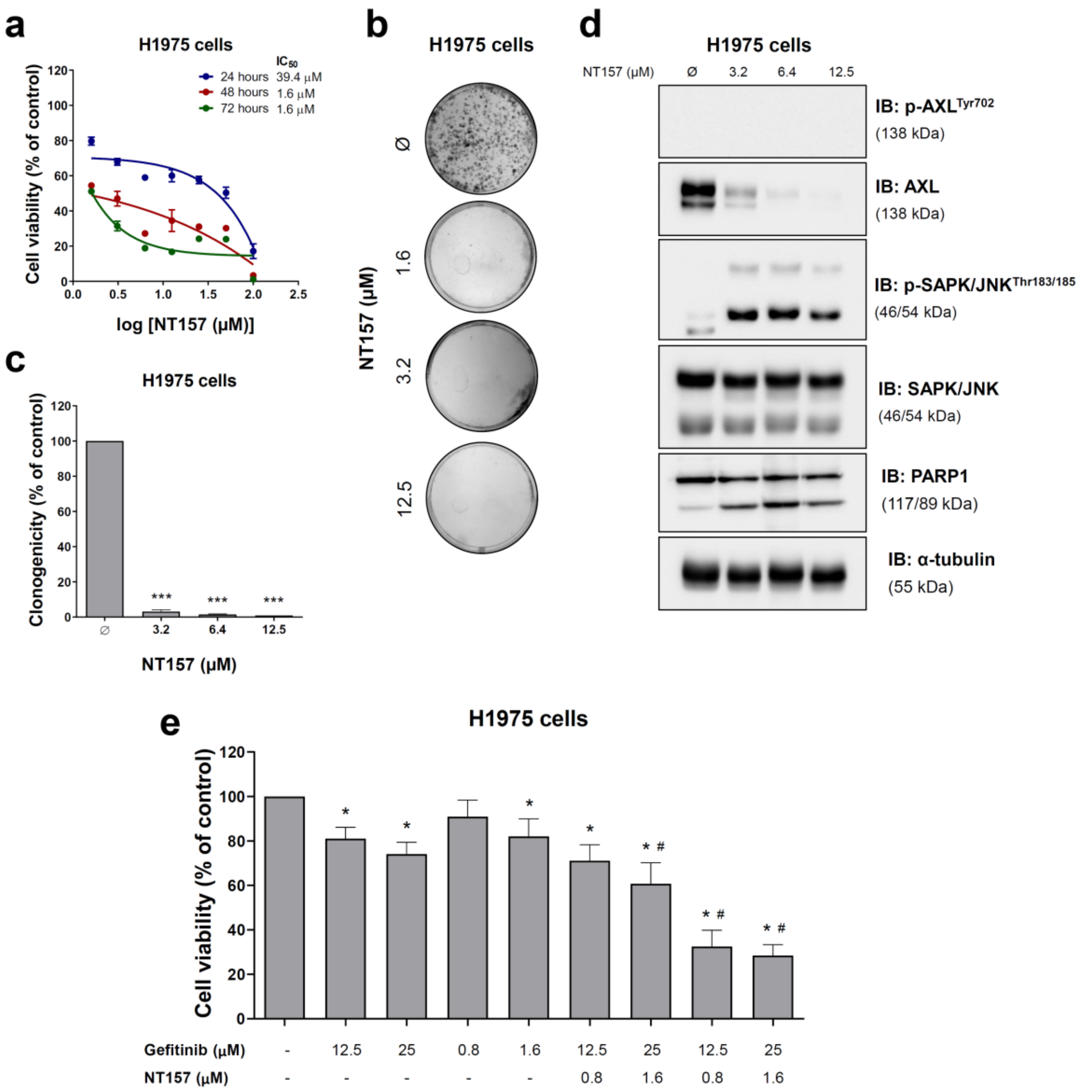

Supplementary Figure 6. NT157 displays antineoplastic activity in H1975, an EGFR-mutated cell line. (a) Dose- and time-response cytotoxicity was evaluated by the sulforhodamine B (SRB) assay. H1975 cells were treated with vehicle (Ø) or different concentrations of NT157 (1.6, 3.2, 6.4, 12.5, 25, 50, and 100 μM) for 24, 48, and 72 h. Values are expressed as the percentage of viable cells for each condition relative to vehicle-treated cells. Results are shown as mean ± SD of at least 3 independent experiments. **(b)** Colony formation of the cells treated with vehicle or NT157 (1.6, 3.2, 6.4, and 12.5 μM) and for 7 days. The bar graph represents the mean ± SD of the relative number of colonies (% of control). ***p < 0.001; ANOVA and Bonferroni post-test. **(c)** Western blot analysis for p-AXL^{Tyr702}, AXL, p-SAPK/JNK$^{Thr183/185}$, SAPK/JNK, and PARP1 in total cell extracts from H1975 cells treated with vehicle or NT157 (3.2, 6.4, and 12.5 μM) for 24 hours. (d) H1974 cells treated with NT157 (0.8 and1.6 μM) and

gefitinib (12.5 and 25 μM) alone or in combination with each other for 48 hours, as indicated. Values are expressed as the percentage of viable cells for each condition relative to vehicle-treated cells. Results are shown as mean ± SD of at least 3 independent experiments. The *p* values are indicated in the graphs; $^{*}p < 0.05$ for gefitinib- and/or NT157-treated cells vs. vehicle-treated cells, $^{\#}p < 0.05$ for gefitinib- or NT157-treated cells versus combination treatment at the corresponding doses; ANOVA and Bonferroni post-test.
